# Evaluation of the Biogenic Amines and Microbial Contribution in Traditional Chinese Sausages

**DOI:** 10.3389/fmicb.2019.00872

**Published:** 2019-05-03

**Authors:** Lu Li, Dian Zou, Liying Ruan, Zhiyou Wen, Shouwen Chen, Lin Xu, Xuetuan Wei

**Affiliations:** ^1^Key Laboratory of Environment Correlative Dietology (Ministry of Education), College of Food Science and Technology, Huazhong Agricultural University, Wuhan, China; ^2^Beijing Advanced Innovation Center for Food Nutrition and Human Health, Beijing Technology and Business University (BTBU), Beijing, China; ^3^Department of Food Science and Human Nutrition, Iowa State University, Ames, IA, United States; ^4^Hubei Collaborative Innovation Center for Green Transformation of Bio-Resources, College of Life Sciences, Hubei University, Wuhan, China; ^5^Carollo Engineers, Inc., Boise, ID, United States

**Keywords:** biogenic amines, Chinese sausages, microbial communities, formation, degradation

## Abstract

Biogenic amines (BAs) in sausages represent a health risk for consumers, and thus investigating the BAs accumulation mechanism is important to control the BAs. In this study, the BAs profiles of 16 typical Chinese sausage samples were evaluated, and 8 kinds of common BAs were detected from different samples. As a whole, the BAs contents of the majority of Chinese sausage samples were within the safe dosage range, except that the total BAs and histamine concentrations of sample HBBD were above the toxic dosage levels. Furthermore, the bacterial and fungal communities of the Chinese sausage samples were investigated by high-throughput sequencing analysis, and *Staphylococcus*, *Bacillus*, *Lactococcus*, *Lactobacillus*, *Debaryomyces*, and *Aspergillus* were identified as the predominant genera. Accordingly, 13 representative strains were selected from the dominant genera, and their BAs formation and degradation properties were evaluated. Finally, the results of fermented meats model experiment indicated that the *Staphylococcus* isolates including *Staphylococcus pasteuri* Sp, *Staphylococcus epidermidis* Se, *Staphylococcus carnosus* Sc1, *Staphylococcus carnosus* Sc2, and *Staphylococcus simulans* Ss could significantly reduce BAs, possessing the potential as the starter cultures to control the BAs in fermented meat products. The present study not only helped to explain the BAs accumulation mechanism in Chinese sausage, but also developed the candidates for potential BAs control in fermented meat products.

## Introduction

Biogenic amines (BAs) are mainly generated from amino acid decarboxylation by food-related microorganisms and are commonly considered as potential toxic nitrogenous substances in foods (Tabanelli et al., 2014; Suzzi and Torriani, 2015; Gardini et al., 2016). The typical BAs include tryptamine, β-phenethylamine, putrescine, cadaverine, histamine, and tyramine, which are the products of microbial decarboxylation of tryptophan, phenylalanine, ornithine, lysine, histidine and tyrosine, respectively (Russo et al., 2016; Li et al., 2018b). In addition to decarboxylation, deimination of agmatine also generates putrescine (Coton et al., 2010). In general, histamine and tyramine are highly toxic among various BA compounds, and tyramine is usually abundant in protein-rich fermented products including cheeses (Mayer and Fiechter, 2018), fermented sausages (Ekici and Omer, 2018), and fish products (Zhang et al., 2013). As a result, the BAs levels have been used as the indicators for food safety and also applied as the quality indexes for good manufacturing practice evaluation (Tasiæ, 2012; Gardini et al., 2016).

Chinese sausages are traditional fermented meat products in China. Due to their characteristic color, texture, and flavor, Chinese sausages have been popular with a long history in China (Wang et al., 2013). However, the Chinese sausages are traditionally manufactured by spontaneous fermentation with negligent quality and safety control (Lu et al., 2010a). In addition, the ripening of Chinese sausages is a complex biochemical process involving interactions of multiple microbial species, which in turn plays an important role in BAs accumulation (Lu et al., 2010b; Chen et al., 2016). While these microbes usually excrete amino acid decarboxylases to produce BAs, they may also degrade the BAs through their native amine oxidases (Xia et al., 2016; Li et al., 2018b). From this perspective, characterizing the microbes in Chinese sausages and evaluating their BAs production and degradation ability is critical to understand the BAs accumulation mechanism, which is ultimately beneficial to control the BAs to guard the quality and safety of Chinese sausages.

The microbial communities can be characterized by cell culturing, colony counting, denaturing gradient gel electrophoresis, and temperature gradient gel electrophoresis (Yang et al., 2016). Compared to these traditional methods, high-throughput sequencing technology can generate thousands of sequences within a short time to cover the complex microbial communities, and it has been applied to characterize microbial diversity of extremely complex environmental ecosystems (Dalmasso et al., 2016; Portillo and Mas, 2016). For example, a previous report evaluated the microbial communities of Chinese sausages by high-throughput sequencing technology, and the *Staphylococcus* was identified as the dominant genus (Wang X. et al., 2018). From a practical point of view, however, it is still a challenge on how to utilize this information of microbial communities for improving the quality and safety of the Chinese sausages. An immediately application could be developing an appropriate starter cultures for better control of BAs in Chinese sausages fermentation process. In this study, we evaluated the BAs contents in 16 typical Chinese sausages samples from different regions in China, and the bacteria and fungi communities were identified by 16S and ITS rDNA gene sequencing analysis, respectively. Furthermore, the BAs production and degradation properties of representative strains were evaluated to elucidate the microbial contribution to BAs accumulation and select beneficial candidates for BAs control.

## Materials and Methods

### Samples and Media

Sixteen Chinese sausage samples collected from different geographical locations in China were used in this work. These samples were labeled as SCMS (Meishan), SCCD (Chengdu), GXWZ (Wuzhou), ZJHZ (Hangzhou), JXJGS (Jinggangshan), GDHP (Huangpu), HNXX (Xiangxi), HLJHEB (Haerbin), HBES (Enshi), ZJJH (Jinhua), JLCC (Changchun), HBBD (Baoding), GZZY (Zunyi), AHXC (Xuancheng), NMGTL (Tongliao), and JSRG (Rugao). Each sample was collected in three replicates, and stored at -20°C for further analysis.

The media used in this study included LB medium (peptone 10 g/L, yeast extract 5 g/L, sodium chloride 10 g/L), MRS medium (peptone 10 g/L, beef extract 8 g/L, yeast extract 4 g/L, glucose 20 g/L, diammonium hydrogen citrate 2 g/L, sodium acetate 5 g/L, K_2_HPO_4_ 2 g/L, MgSO_4_ 0.2 g/L, MnSO_4_ 0.04 g/L, Tween 80 1 g/L, pH = 5.7), and SDB medium (peptone 10 g/L, glucose 20 g/L, pH = 5.6). Agar (1.5 wt%) was added to prepare the solid medium.

### Microbial Community Analysis

The genomic DNA was extracted from the Chinese sausage samples with the E.Z.N.A Soil DNA kit (OMEGA, United States) following the manufacturer’s instructions. The purity of the genomic DNA was confirmed by the subsequent pyrosequencing analysis with 1% agarose gel electrophoresis. Primers 338F (ACTCCTACGGGAGGCAGCA) and 806R (GGACTACHVGGGTWTCTAAT) were designed according to the V3–V4 region of bacterial 16S rRNA gene. Primers ITS1F (CTTGGTCATTTAGAGGAAGTAA) and 2043R (GCTGCGTTCTTCATCGATGC) were designed based on the ITS1F-ITS2 region of the fungal internal transcribed spacer (ITS). After PCR and purification, a DNA library was constructed and run on the Miseq Illumina platform at Majorbio Bio-Pharm Technology Co., Ltd. (Shanghai, China).

Sequencing data was analyzed using the software of Trimmomatic and FLASH. Community estimators were calculated and analyzed using Mothur version v.1.30.1^1^, including richness estimators, Chao1 index, diversity estimators, and Shannon index. The number of operational taxonomic units (OTUs) (with >97% sequences similarity being defined as one OTU) was obtained by Usearch program (version 7.1) using furthest neighbor algorithm and established the phylogenetic tree with the relative abundances of OTUs. Taxonomy was assigned by the Silva Database Project classifier (Quast et al., 2013).

### Isolation of Strains From Chinese Sausage Samples

Chinese sausage samples (5 g) were crushed and added with 45 mL sterile water. The mixtures were incubated at 37°C for 40 min in a rotatory shaker with 140 rpm. Samples were then serially diluted (10^-1^ to 10^-6^) with the sterile water. For each dilution, 200 μL sample solution was, respectively, plated onto LB plates, MRS plates with 20 g/L CaCO_3_ and SDB plates with 50 mg/L rifampicin. LB plates were incubated at 37°C for 24 h. SDB plates were cultured at 37°C for 48–72 h (depending on the strains), and MRS agar plates were incubated at 37°C for 48 h. And the colonies with different forms were selected for further analysis.

### Strains Identification

Genomic DNA of bacteria was extracted using Gen-EluteTM Kit (Tiangen Biotech Co., Ltd., Beijing, China) following the manufacturer’s protocol, and the genomic DNA of fungi was extracted using a SDS-based DNA extraction method described previously (Zhou et al., 1996). The 16S rDNA sequence was amplified using the universal primers of 27f (AGAGTTTGATCMTGGCTCAG) and 1492r (CTACGGCTACCTTGTTACGA), and the ITS fragment was amplified with the universal primers ITS1 (TCCGTAGGTGAACCTGCGG) and ITS4 (GCATATCAATAAGCGGA). The PCR procedure followed the protocols reported previously (Li et al., 2018a,b). The PCR amplicons were sequenced and analyzed using the Blastn program^2^.

### Detection of the Genes Related to BAs

PCR amplification were performed to confirm the presence of BAs-related genes of histidine decarboxylase (*hdcA*), tyrosine decarboxylase (*tyrdc*), ornithine decarboxylase (*odc*), agmatine deiminase (*aguA* and *aguD*) and lysine decarboxylase (*ldc*). The primers used in this study were listed in Supplementary Table S1. The PCR procedure followed the protocols reported previously (Guarcello et al., 2016; Li et al., 2018b). The PCR products were analyzed by electrophoresis on a 0.8% agarose gel and revealed under UV after staining with ethidium bromide.

### Evaluation of BAs Production and Degradation Properties

The BAs production properties of different strains were characterized by assessing the biotransformation of precursors to corresponding BAs. The strains were cultured in 5 mL LB medium (*Bacillus* and *Staphylococcus*), MRS medium (*Enterococcus* and *Lactobacillus*) or SDB medium (*Candida*), and all the media were added with 1 g/L of histidine, tyrosine, tryptophan, phenylalanine, ornithine monohydrochloride, lysine, or agmatine sulfate salt. The BAs concentrations after 48 h incubating were determined. To evaluate the BAs degradation properties, the strains were cultured at 37°C for 12–24 h and then collected by centrifugation at 6000 ×*g* for 5 min. The cell pellets were washed with 0.05 mol/L phosphate buffer (pH = 7), re-suspended to reach OD600 at 0.8 in phosphate buffer (0.05 mol/L) containing 100 mg/L of histamine, tyramine, tryptamine, β-phenethylamine, putrescine, cadaverine, spermidine and spermine. The cell suspension was then cultured at 37°C for 48 h and the residual BAs in the suspension was determined. The phosphate buffer without cell pellets was applied as control. The BA-degradation rate was calculated as

M=[(A−B)/A]×100%,

where *M* is the BAs degradation percentage, *A* and *B* are initial and residual BAs concentrations, respectively (Li et al., 2018a,b).

### Fermented Meat Model Analysis

The model fermented meat was constructed to evaluate the BAs-controlling properties of as-selected strains. In brief, the cells were inoculated into corresponding broth at 37°C for 12 h to reach OD_600_ of 4.0. The culture was used as the seed (5%, v/w) to be inoculated into a 50 mL sterilized flask with 10 g of fresh pork slices containing 2% salt, 1% glucose and 4% sucrose. The microbe-meat mixture was incubated at 37°C for 7 days to analyze the total BAs, and the meat inoculated with equal volume of sterilized water was used as the control.

### Determination of BAs Concentrations

The BAs were extracted and pretreated according to the methods described previously (Li et al., 2018a,b). The BAs were analyzed using an Agilent 1260 HPLC. The separation of the analytes was achieved with a Zorbax Eclipse XDB-C18 (4.6 mm × 250 mm, 5 μm) column in an oven at 30°C. The injection volume was 10 μL. Chromatograms were analyzed at 254 nm. Two reservoirs containing (A) ultrapure water and (B) acetonitrile were used to provide elution solution. The elution flow rate was 1.0 mL/min. The gradient elution program was 0–3 min, *A*/*B* = 1:1; 3–20 min, *A*/*B* = 1:1 to *A*/*B* = 1:9; 20–29 min, *A*/*B* = 1:9; 29–32 min, *A*/*B* = 1:9 to *A*/*B* = 1:1; 32–35 min, *A*/*B* = 1:1.

### Statistical Analysis

All analysis experiments were conducted at least three replicates. The difference significance was analyzed by one-way ANOVA method using the statistical software SPSS 20.0, and mean values were compared by Tukey’s HSD test at 5%.

## Results

### BAs Contents in Chinese Sausage Samples

The BAs contents in different Chinese sausage samples were shown in Table 1. Among the sixteen Chinese sausage samples, eight BAs were detected, including tryptamine, β-phenethylamine, putrescine, cadaverine, histamine, tyramine, spermidine, and spermine. Putrescine, cadaverine, tyramine, spermidine, and spermine were the dominant BAs, and they existed in the majority of Chinese sausage samples. Tryptamine, β-phenethylamine and histamine were detected in just several samples. Among sixteen Chinese sausage samples, the total BAs contents were different with each other. HBBD sample had the highest content of the total BAs (1417.57 mg/kg), as well as the highest concentration of tryptamine, β-phenethylamine, putrescine, cadaverine, histamine, or tyramine. On the contrary, ZJHZ sample showed the lowest value of the total BAs concentration (32.24 mg/kg), with only spermidine and spermine detected.

**Table 1:** The BAs contents of Chinese sausage samples.

Chinese sausage sample	Tryptamine (mg/kg)	β-Phenethylamine (mg/kg)	Putrescine (mg/kg)	Cadaverine (mg/kg)	Histamine (mg/kg)	Tyramine (mg/kg)	Spermidine (mg/kg)	Spermine (mg/kg)	Total (mg/kg)
SCMS	16.71 ± 1.47 b	ND	171.75 ± 11.94 c	26.46 ± 1.84 de	ND	115.61 ± 24.53 c	6.35 ± 0.83 bcde	16.15 ± 3.62 bcde	353.03 ± 44.23 c
SCCD	ND	ND	2.57 ± 0.12 a	20.20 ± 0.55 cd	1.19 ± 0.05 a	19.11 ± 0.97 ab	4.19 ± 0.06 abcd	14.17 ± 0.47 abcd	61.43 ± 2.22 a
GXWZ	1.61 ± 0.90 a	3.24 ± 0.68	1.09 ± 0.52 a	2.25 ± 0.37 a	ND	31.3 ± 7.76 ab	23.50 ± 3.02 h	23.27 ± 2.39 ef	86.26 ± 15.64 a
ZJHZ	ND	ND	ND	ND	ND	ND	10.58 ± 1.55 g	21.66 ± 2.95 de	32.24 ± 4.5 a
JXJGS	ND	ND	5.64 ± 0.42 ab	162.67 ± 8.79 h	ND	34.50 ± 2.68 ab	7.10 ± 0.28 def	19.93 ± 0.77 cde	229.84 ± 12.94 b
GDHP	ND	ND	ND	42.84 ± 2.85 f	ND	16.66 ± 0.19 a	6.93 ± 0.60 cdef	16.02 ± 1.75 bcde	82.45 ± 5.39 a
HNXX	ND	ND	3.17 ± 1.02 a	55.33 ± 5.18 g	ND	22.13 ± 2.92 ab	3.79 ± 0.32 abc	18.47 ± 1.42 cde	102.89 ± 10.86 a
HLJHEB	ND	ND	ND	15.54 ± 1.51 bcd	ND	12.47 ± 1.97 a	2.44 ± 0.34 a	14.02 ± 3.62 abc	44.47 ± 7.44 a
HBES	ND	ND	0.47 ± 0.22 a	30.8 ± 2.91 e	4.23 ± 2.99 a	12.99 ± 2.38 a	3.27 ± 0.22 ab	21.39 ± 2.57 cde	73.24 ± 11.29 a
ZJJH	ND	ND	ND	7.22 ± 0.72 ab	ND	2.38 ± 0.14 a	9.99 ± 0.59 fg	19.81 ± 1.47 cde	39.4 ± 2.92 a
JLCC	ND	ND	2.16 ± 0.36 a	15.45 ± 1.83 bcd	ND	54.79 ± 6.43 abc	10.06 ± 1.20 fg	36.53 ± 4.38 g	118.99 ± 14.2 a
HBBD	22.49 ± 1.58 c	8.22 ± 4.34	277.07 ± 5.65 d	670.92 ± 8.4 j	209.64 ± 7.84 b	209.57 ± 83.68 d	4.18 ± 0.78 abcd	15.50 ± 4.6 bcd	1417.59 ± 116.87 d
GZZY	ND	ND	16.51 ± 2.12 b	177.62 ± 4.3 i	4.89 ± 0.28 a	85.52 ± 1.36 bc	8.98 ± 0.26 efg	35.82 ± 1.07 g	329.34 ± 9.39 c
AHXC	ND	ND	3.55 ± 0.68 a	11.85 ± 1.09 abc	ND	12.66 ± 0.76 a	9.21 ± 0.39 efg	10.72 ± 0.44 ab	47.99 ± 3.36 a
NMGTL	ND	ND	3.08 ± 0.68 a	9.32 ± 0.29 abc	ND	5.30 ± 0.27 a	6.84 ± 1.81 cdef	29.84 ± 2.01 fg	54.38 ± 5.06 a
JSRG	ND	ND	ND	14.48 ± 2.91 bc	ND	12.52 ± 2.39	4.77 ± 0.29 abcd	7.48 ± 0.54 a	39.25 ± 6.13 a

*Different letters (a, b, c, d, e, f, g, h, i, j) indicate significantly different means at P < 0.05 [analysis of variance (ANOVA)].*

### Microbial Communities in Chinese Sausage Samples

After the quality control, a total of 1,351,248 high-quality 16S rRNA gene sequences and 1,490,880 high-quality ITS gene sequences were recovered from sixteen sausage samples. The Chao richness estimator and Shannon index were calculated based on 3% genetic distance for the samples, and the results are shown in Table 2. The Good’s estimator of coverage was 99% for all samples, indicating that the majority of bacterial and fungal phylotypes were detected. The maximum number of bacterial OTUs was found in JSRG sample, and the highest fungal OTUs number was obtained in HBBD sample. Both bacterial and fungal OTUs were the lowest in NMGTL sample.

**Table 2:** The operational taxonomic units (OTUs) for each Chinese sausage sample.

Chinese sausage sample	Bacteria				Fungi			
	Observed OTUs	Chao1	Shannon	Good’s coverage (%)	Observed OTUs	Chao1	Shannon	Good’s coverage (%)
SCMS	288 ± 54	352 ± 40	2.56 ± 0.58	99	165 ± 32	193 ± 47	1.47 ± 0.34	99
SCCD	145 ± 9	215 ± 16	1.63 ± 0.21	99	32 ± 4	63 ± 13	0.08 ± 0.06	99
GXWZ	133 ± 6	186 ± 32	1.35 ± 0.47	99	214 ± 16	216 ± 17	3.79 ± 0.05	99
ZJHZ	256 ± 25	292 ± 18	1.92 ± 0.14	99	209 ± 17	216 ± 15	3.65 ± 0.12	99
JXJGS	190 ± 19	276 ± 62	2.36 ± 0.19	99	98 ± 25	103 ± 28	2.94 ± 0.45	99
GDHP	204 ± 15	266 ± 33	2.78 ± 0.03	99	220 ± 28	234 ± 29	3.28 ± 0.44	99
HNXX	240 ± 9	306 ± 20	3.05 ± 0.23	99	197 ± 27	207 ± 31	2.90 ± 0.35	99
HLJHEB	305 ± 48	381 ± 36	2.17 ± 0.29	99	218 ± 9	220 ± 8	3.40 ± 0.42	99
HBES	280 ± 24	342 ± 28	3.05 ± 0.14	99	186 ± 5	200 ± 13	3.50 ± 0.22	99
ZJJH	189 ± 1	250 ± 12	1.94 ± 0.26	99	89 ± 13	93 ± 9	1.54 ± 0.02	99
JLCC	294 ± 29	338 ± 46	2.70 ± 0.24	99	216 ± 6	230 ± 4	3.69 ± 0.07	99
HBBD	185 ± 20	262 ± 11	2.40 ± 0.09	99	240 ± 25	247 ± 25	3.84 ± 0.18	99
GZZY	191 ± 17	240 ± 27	2.33 ± 0.36	99	146 ± 19	160 ± 22	0.58 ± 0.24	99
AHXC	315 ± 23	371 ± 30	3.62 ± 0.06	99	129 ± 19	139 ± 25	2.35 ± 0.40	99
NMGTL	45 ± 18	74 ± 28	0.14 ± 0.03	99	17 ± 12	41 ± 17	0.04 ± 0.05	99
JSRG	356 ± 5	402 ± 11	3.38 ± 0.05	99	140 ± 17	180 ± 10	1.62 ± 0.00	99

**FIGURE 1:**
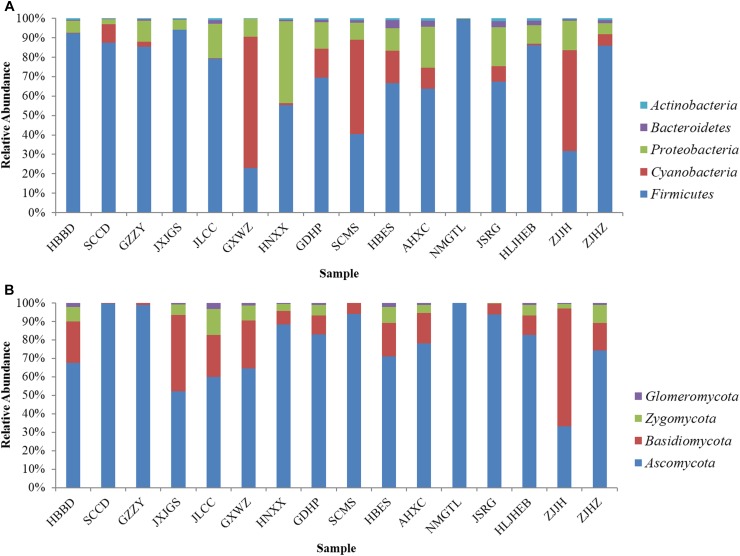
Microbial community in Chinese sausage samples at the phylum level. **(A)** Bacteria, **(B)** fungi.

**FIGURE 2:**
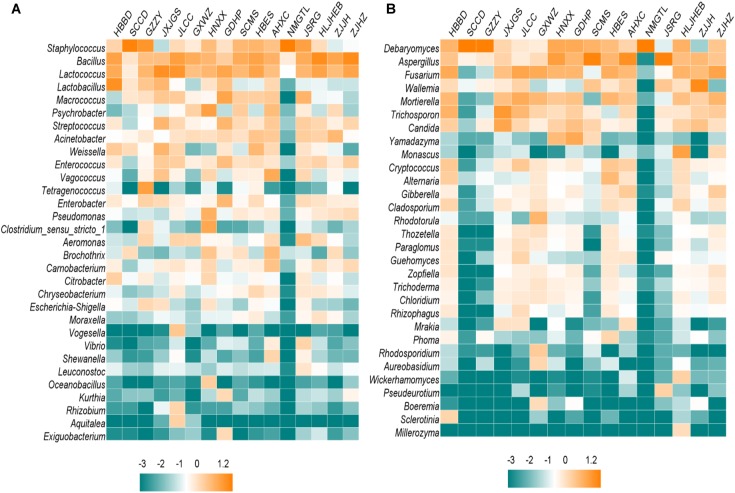
Heatmap of Chinese sausage samples at the genus level. The color intensity of each panel is proportional to the OTU abundance. **(A)** Bacteria, **(B)** fungi.

Based on 16S rRNA gene sequence, *Firmicutes* was predominant bacterial phylum in most samples, particularly in NMGTL with up to 99.82% of the total sequences (Figure 1A). *Cyanobacteria* was the main phylum in GXWZ, SCMS, and ZJJH samples, accounting for 67.60, 48.60, and 51.84% to total phylum, respectively. At the genus level, *Staphylococcus* existed in all sausage samples and was particularly high in SCCD and NMGTL samples, representing up to 80.06 and 98.40% of total genus, respectively (Figure 2A). *Bacillus* and *Lactococcus* were also widely distributed in sausage samples. The genera of *Lactobacillus*, *Macrococcus*, *Psychrobacter*, *Tetragenococcus*, and *Pseudomonas* were predominant in some samples.

As to fungi, *Ascomycota* was the predominant phylum in most sausage samples, while *Basidiomycota* was only dominant in ZJJH sample (Figure 1B). *Ascomycota* occupied 99.70, 98.84, and 99.98% in the sample of SCCD, GZZY, and NMGTL, respectively. The heatmap revealed differences in fungal genera among different sausage samples (Figure 2B). *Debaryomyces* was the prevailing genus in SCCD, GZZY, and NMGTL. *Aspergillus* was the predominant genus in the sample of SCMS, AHXC, and JSRG. *Wallemia* was high in ZJJH sample, representing up to 58.31% of the total sequences, much higher than that of the other samples.

The relationships between microbial communities and BAs were also evaluated by spearman analysis (Supplementary Figure S1). The genera of *Lactobacillus*, *Weissella*, and *Citrobacter* showed positive correlation (*P* < 0.05) with tryptamine, β-phenethylamine, putrescine, cadaverine, histamine and tyramine. The *Candida* genus was positively correlated (*P* < 0.05) with β-phenethylamine, cadaverine and histamine. The genera such as *Enterococcus* and *Streptococcus*, were commonly reported as BA-producers, while spearman analysis showed that they had no relationship with BAs formation. Due to the complexity of microbial communities, the correlation analysis might not reveal the accurate relationship between microbial communities and BAs. Therefore, it is necessary to isolate the representative strains from the dominant communities to evaluate their capabilities to produce and degrade BA, which will be more accurate to reflect the relationships between main strains and BAs.

### Contribution of the Main Microbes on BAs

The sausages sample HBBD with the highest BAs content probably contained the main BAs-producing strains, and the sample ZJHZ with the lowest BAs content might possess the efficient BAs-degrading strains. Therefore, these two samples were used to select the target strains. Meanwhile, *Staphylococcus*, *Bacillus*, and *Lactobacillus* were major genera in most sausages samples, and *Staphylococcus* species were commonly used as the starter cultures in fermented meat products. Therefore, we mainly selected the isolates belonging to *Staphylococcus*, *Bacillus*, and *Lactobacillus*. We obtained 13 isolates, including *Staphylococcus pasteuri* (1 isolate), *Staphylococcus epidermidis* (1 isolate), *Staphylococcus carnosus* (2 isolates), *Staphylococcus simulans* (1 isolate), *Bacillus subtilis* (1 isolate), *Bacillus amyloliquefaciens* (1 isolate), *Bacillus pumilus* (1 isolate), *Enterococcus faecium* (3 isolates), *Lactobacillus curvatus* (1 isolate), and *Candida metapsilosis* (1 isolate) (Table 3).

**Table 3:** 16S rDNA and ITS sequences similarities of isolated strains with representative strains.

Isolates	Closest strains	Identities (%)	Accession number
Sp	*S*. *pasteuri* HN-35	99%	KT003275.1
Se	*S*. *epidermidis* 14F	99%	KC920585.1
Sc1	*S*. *carnosus* JX-1	99%	MH445557.1
Sc2	*S*. *carnosus* CIP103274	99%	NR_116433.1
Ss	*S*. *simulans* MR1	99%	CP015642.1
Bs	*B*. *subtilis* J2	99%	KT957306.1
Ba	*B*. *amyloliquefaciens* 1Y018	100%	JQ229807.1
Bp	*B*. *pumilus* WM2	99%	KY085970.1
Ef1	*E*. *faecium* CAU-200	99%	MF369859.1
Ef2	*E*. *faecium* KLAB2	99%	KM497516.1
Ef3	*E*. *faecium* KC11	99%	KM497512.1
Lc	*L*. *curvatus* GAN	99%	MH194558.1
Cm	*C*. *metapsilosis* IFM52611	99%	LC389302.1

*Identities: the 16S rDNA and ITS sequences identities of isolated strains with representative strains. Accession number: the GenBank accession number of the 16S rDNA or ITS sequences of the representative strains.*

**FIGURE 3:**
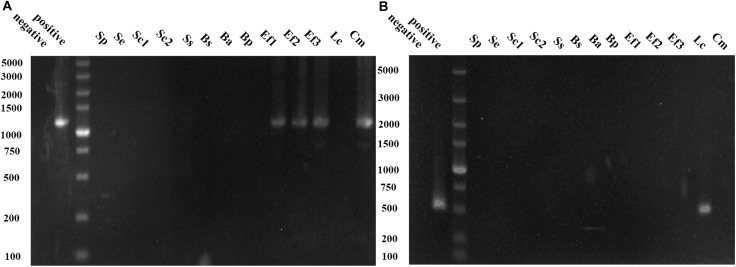
PCR results for the genes involved in biogenic amine (BAs) production. **(A)** PCR products using the primer pair TD2/TD5 for identification of tyrosine decarboxylase gene. **(B)** PCR products using the primer pair ODF/ODR for identification of ornithine decarboxylase gene.

Common BAs-forming genes in 13 isolates were detected to evaluate their potential BAs-producing abilities, and their capabilities for biotransformation of amino acid precursor to corresponding BA were also measured. Figure 3A showed *C. metapsilosis* Cm and three *E. faecium* isolates were positive in tyrosine decarboxylase gene detection, and they also showed strong tyramine-producing abilities (Table 4). The ornithine decarboxylase gene was only detected in *L. curvatus* Lc (Figure 3B), which agreed with the fact that just *L. curvatus* Lc had the capacity to produce putrescine from ornithine (Table 4). None of the isolates was positive in histidine decarboxylase gene (*hdcA*), lysine decarboxylase gene (*ldc*) and agmatine deiminase gene (*aguA* and *aguD*) using primers HdC1/HdC2, Cad2F/Cad2R, CadAf/CadAr, AgmSq1/AgmSq2, and AgD1/AgD2. Accordingly, most isolated strains showed no ability to produce histamine, cadaverine and putrescine from histidine, lysine and agmatine, respectively, and just low concentrations were detected from several strains. These results indicated that unknown amine acid decarboxylases or isoenzymes with low activities might exist in some strains, which will be further investigated in the future. On the other hand, more than 60% of the strains produced tryptamine and β-phenethylamine. Among the 13 isolates, *S. epidermidis* Se and *S. simulans* Ss showed no capability to generate BAs, and *S. pasteuri* Sp, *B. subtilis* Bs, and *B. amyloliquefaciens* Ba produced low concentrations of BAs, with high contents of BAs observed in the rest isolates.

**Table 4:** The BA-producing abilities of the isolated strains with corresponding precursor.

Strain	Tryptamine (mg/L)	β-Phenethylamine (mg/L)	Putrescine (Ornithine monohydrochloride) (mg/L)	Putrescine (Agmatine sulfate salt) (mg/L)	Cadaverine (mg/L)	Histamine (mg/L)	Tyramine (mg/L)	Total (mg/L)
Sp	ND	33.69 ± 6.69 ab	ND	ND	ND	ND	ND	33.69 ± 6.69 a
Se	ND	ND	ND	ND	ND	ND	ND	ND
Sc1	155.83 ± 6.25 c	428.33 ± 31.03 f	ND	ND	ND	ND	18.35 ± 1.24 a	602.51 ± 38.52 c
Sc2	94.50 ± 3.35 b	166.95 ± 61.51 cd	ND	ND	ND	ND	ND	261.45 ± 64.86 ab
Ss	ND	ND	ND	ND	ND	ND	ND	ND
Bs	ND	23.53 ± 1.41 a	ND	ND	ND	ND	ND	23.53 ± 1.41 a
Ba	ND	20.93 ± 2.31 a	ND	0.41 ± 0.17 a	ND	ND	ND	21.34 ± 2.48 a
Bp	274.04 ± 13.69 d	100.44 ± 3.30 bc	ND	ND	ND	ND	ND	374.48 ± 16.99 bc
Ef1	10.05 ± 0.12 a	241.79 ± 3.55 de	ND	7.81 ± 1.82 b	5.74 ± 0.61 b	18.72 ± 1.18 b	1004.38 ± 33.87 bc	1288.49 ± 41.15 e
Ef2	6.55 ± 2.62 a	96.86 ± 14.73 abc	ND	6.89 ± 1.69 b	ND	21.29 ± 0.89 b	1218.89 ± 49.39 c	1350.48 ± 69.29 e
Ef3	11.14 ± 0.57 a	261.17 ± 41.98 e	ND	ND	6.14 ± 1.68 b	19.68 ± 0.56 b	1081.22 ± 85.36 bc	1379.35 ± 130.15 e
Lc	6.22 ± 0.71 a	ND	172.25 ± 2.71	12.12 ± 1.10 c	0.67 ± 0.34 a	ND	ND	191.26 ± 4.86 ab
Cm	7.01 ± 0.07 a	57.33 ± 12.17 ab	ND	ND	ND	9.56 ± 5.35 a	817.84 ± 240.38 b	891.74 ± 257.97 d

*Different letters (a, b, c, d, e, f) indicate significantly different means at P < 0.05 [analysis of variance (ANOVA)].*

The 13 isolates were tested for their capabilities of degrading tryptamine, β-phenethylamine, putrescine, cadaverine, histamine, tyramine, spermidine, and spermine. Most isolates were capable of degrading eight common BAs with different efficiencies (Table 5). As a whole, all the screened strains were able to completely degrade spermine, and they also showed high degradation abilities of putrescine. Among 13 isolates, all the *Staphylococcus* isolates exhibited relatively high degradation abilities for each BA.

**Table 5:** Profiles of BA-degradation rates of selected strains.

Strain	Tryptamine (%)	β-Phenethylamine (%)	Putrescine (%)	Cadaverine (%)	Histamine (%)	Tyramine (%)	Spermidine (%)	Spermine (%)
Sp	20.60 ± 3.42 cd	16.87 ± 3.12 abc	70.01 ± 5.16 a	23.58 ± 4.57 bc	42.03 ± 5.37 de	29.24 ± 9.45 d	31.72 ± 4.59 d	100
Se	24.54 ± 2.35 d	28.24 ± 6.95 c	63.84 ± 2.09 a	22.47 ± 4.08 bc	43.23 ± 5.69 de	23.25 ± 5.35 cd	22.44 ± 5.72 cd	100
Sc1	21.29 ± 2.22 d	24.08 ± 2.41 bc	69.72 ± 3.17 a	20.43 ± 2.78 abc	41.46 ± 4.68 de	19.64 ± 4.83 bcd	19.21 ± 4.67 bc	100
Sc2	22.46 ± 2.02 d	25.49 ± 1.97 bc	68.97 ± 3.80 a	13.22 ± 3.17 a	32.37 ± 1.80 cd	18.71 ± 2.59 abcd	19.29 ± 2.06 bc	100
Ss	18.32 ± 1.25 bcd	16.47 ± 1.93 abc	63.98 ± 2.05 a	17.58 ± 1.03 abc	38.07 ± 4.72 de	14.55 ± 1.75 abc	13.60 ± 0.44 abc	100
Bs	7.50 ± 1.34 a	7.48 ± 1.63 a	65.69 ± 2.93 a	12.07 ± 0.98 a	15.37 ± 2.64 ab	7.45 ± 0.92 a	7.57 ± 1.51 a	100
Ba	20.66 ± 3.03 cd	19.52 ± 3.56 abc	69.50 ± 4.19 a	24.33 ± 3.63 bc	11.08 ± 2.3 a	21.62 ± 2.75 bcd	20.91 ± 2.18 bc	100
Bp	11.95 ± 2.72 ab	14.83 ± 0.97 ab	68.09 ± 2.24 a	16.48 ± 3.11 ab	5.76 ± 1.94 a	13.63 ± 2.80 abc	12.97 ± 1.19 abc	100
Ef1	0.00	0.00	60.59 ± 5.02 a	0.00	36.15 ± 2.20 cde	0.00	0.00	100
Ef2	12.59 ± 0.06 abc	11.09 ± 0.74 a	67.48 ± 4.68 a	17.57 ± 2.66 abc	39.34 ± 2.92 de	13.82 ± 0.44 abc	16.93 ± 0.56 abc	100
Ef3	19.24 ± 4.06 bcd	19.56 ± 3.61 abc	70.67 ± 2.56 a	25.82 ± 3.52 c	47.76 ± 7.45 e	17.94 ± 2.63 abcd	20.26 ± 4.92 bc	100
Lc	0.00	0.00	63.23 ± 6.22 a	0.00	0.00	0.00	0.00	100
Cm	21.43 ± 5.25 d	59.01 ± 9.91 d	67.38 ± 1.60 a	16.80 ± 3.13 abc	24.34 ± 3.68 bc	10.80 ± 3.03 ab	11.17 ± 3.70 ab	100

*Different letters (a, b, c, d, e) indicate significantly different means at P < 0.05 [analysis of variance (ANOVA)].*

### Effects of *Staphylococcus* Strains on BAs Accumulation in Model Fermented Meat

**FIGURE 4:**
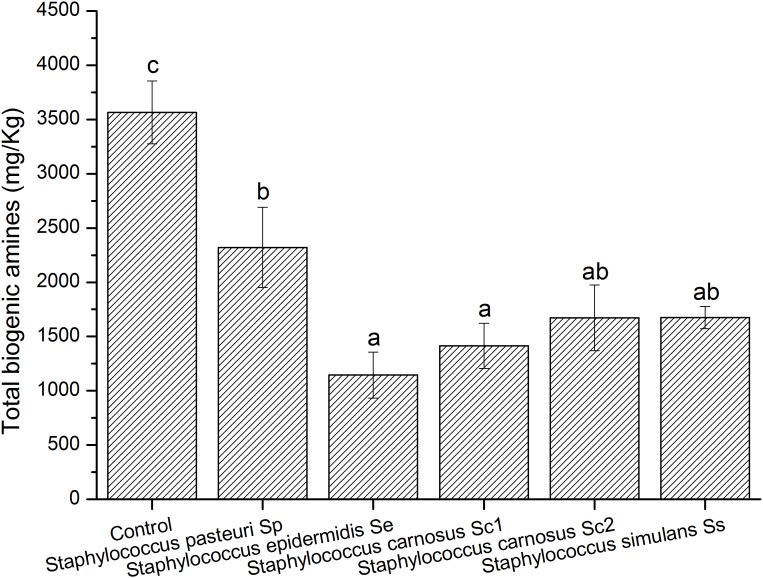
Biogenic amine contents in model fermented meat product inoculated with different strains. Different letters (a, b) indicate significantly different means at *P* < 0.05.

Fermentation of sausages is a complex process involving various microbes, thus we constructed a spontaneous fermentation meat model to investigate effects of the strains on BAs control. Above results showed that all the *Staphylococcus* isolates showed high BAs-degradation abilities (Table 4). Moreover, the *Staphylococcus* strains were commonly used as the starter cultures in fermented meat products (Blaiotta et al., 2010; Semedo-Lemsaddek et al., 2016). Therefore, all the *Staphylococcus* isolates were selected for the model fermented meat experiment to evaluate their potential to reduce the BA contents. As shown in Figure 4, the control sample without inoculation of *Staphylococcus* strain showed a high content of the total BAs (3565 mg/kg), which was probably due to the native microbes in meat. And the individual content of BA found in the meat model were indicated in Supplementary Table S2. Compared with the control, the BAs contents of the samples enriched with *S. pasteuri* Sp, *S. epidermidis* Se, *S. carnosus* Sc1, *S*. *carnosus* Sc2, or *S. simulans* Ss, were reduced (Figure 4). In particular, *S. epidermidis* Se, *S. carnosus* Sc1, *S*. *carnosus* Sc2, and *S. simulans* Ss revealed relatively high abilities of inhibiting the BAs accumulation in model fermented meat.

## Discussion

Excess intake of BAs can cause various harmful effects, for example that histamine can lead to nausea, headache, hot fushes and skin rashes, and tyramine, β-phenylethylamine and tryptamine can cause migraine and hypertensive crises (Suzzi and Gardini, 2003; Del Rio et al., 2017; Doeun et al., 2017). However, BAs have been reported in many fermented foods, and thus evaluating the BAs contents is particularly valuable to indicate the food safety. In this study, eight common BAs were detected in sixteen Chinese sausage samples, and putrescine, cadaverine, tyramine, spermidine, and spermine existed in most of the samples, which was similar with that of previous reports (González-Fernández et al., 2003; Lu et al., 2015). In comparison, putrescine and tyramine were found as major BAs in Italian Sausages (Salami) (Mayr and Schieberle, 2012), and putrescine and cadaverine were at high levels in Danish sausage (Doeun et al., 2017). The total BAs level higher than 1,000 mg/kg in food was considered harmful for human health (Kim et al., 2012). In comparison, the majority of Chinese sausage samples were under the safe level, just the BAs concentration of HBBD sample exceeded the toxic dose (1,000 mg/kg), especially that the histamine content was much higher than toxic level (50 mg/kg) suggested by the United States Food and Drug Administration (Yang et al., 2014). Besides, the concentrations of tyramine in SCMS and HBBD samples were over 100 mg/kg, which might negatively affect human health. Therefore, it is necessary to monitor and control the BAs amounts in Chinese sausages.

Generally speaking, the accumulated BAs in fermented foods mainly depend on two sides, including the BAs formation by amino acids decarboxylases and the BAs degradation by amine oxidases, which were significantly affected by complex microbial communities (Gu et al., 2018; Li et al., 2018a). The marked variable BAs contents in Chinese sausage samples were probably due to the different microbial compositions. Therefore, evaluating the microbial communities and their contribution on BAs formation is particularly valuable to control the BAs in Chinese sausage samples. High-throughput sequencing was used to assess the microbial communities in the sixteen Chinese sausage samples. *Firmicutes* and *Ascomycota* were the predominant phylum in bacteria and fungi, respectively. At the genus level, *Staphylococcus*, *Bacillus*, *Lactococcus*, and *Lactobacillus* were the dominant bacterial genera in most samples. Similar results were also reported that *Staphylococcus* and lactic acid bacteria were the representative genera in fermented meat products (Freiding et al., 2011; Oki et al., 2011; Woraprayote et al., 2016; Wang X. et al., 2018). *Debaryomyces* and *Aspergillus* were the predominant fungal genera in some sausage samples, and these fungi were also reported as the major genera in other meat products (Sørensen et al., 2008; Asefa et al., 2009).

According to the microbial community results, we screened thirteen representative strains from two typical sausage samples with significantly different BAs contents, including *Staphylococcus*, *Bacillus*, *Enterococcus*, *Lactobacillus*, and *Candida*, which were commonly reported in fermented meat products (Vilar et al., 2000; Barrière et al., 2001; Hugas et al., 2003; Matarante et al., 2004; Naidoo and Lindsay, 2010; Rai et al., 2010; Freiding et al., 2011; Zaman et al., 2011; Wang H. et al., 2018). The BA formation abilities of these selected strains were evaluated by gene and biotransformation analysis. Most gene analysis results agreed with biotransformation results. For example, *E. faecium* Ef1, Ef2, Ef3 and *C. metapsilosis* Cm possessed the tyrosine decarboxylase gene, as well as the high tyramine-generating abilities. The ornithine decarboxylase gene was just detected in *L. curvatus* Lc, and identical conclusion was also obtained in biotransformation analysis, which was consistent with a previous study (Li et al., 2018b). Exceptionally, several strains produced low level BA without corresponding decarboxylase gene detected, indicating that unknown amine acid decarboxylase might exist in these strains. All the selected *E. faecium* also showed high capacity of producing β-phenylethylamine, which was similar with previous researches (Marcobal et al., 2006; Li et al., 2018a). These strains with high BAs production properties might be responsible for the accumulation of BAs in Chinese sausage samples. In addition, the BAs degradation abilities of 13 isolates were also analyzed, and the *Staphylococcus* isolates showed relatively high degradation abilities for all BAs. The results indicated that it was possible design of microbial-based solutions to reduce BAs content in fermented food (Capozzi et al., 2012; Latorre-Moratalla et al., 2012; Ladero et al., 2016). Meanwhile, *Staphylococcus* were usually used as the starter cultures for sausages fermentation (Aro et al., 2010; Tabanelli et al., 2012; Aida et al., 2013). Thus, effects of *Staphylococcus* isolates on BAs accumulation were accessed in model fermented meat to select the potential starter culture for BA control. Interestingly, inoculation of *S. pasteuri* Sp, *S. epidermidis* Se, *S. carnosus* Sc1, *S*. *carnosus* Sc2, and *S. simulans* Ss could significantly reduce the BAs accumulation, and these *Staphylococcus* strains could be used as potential candidates for BAs control in fermented meat products. In previous studies, *S. carnosus* FS19 and *Staphylococcus xylosus* No. 0538 were reported as starter cultures to control the BAs accumulation in fermentation of meat products (Mah and Hwang, 2009; Zaman et al., 2011), and this study also developed novel strains as potential starter cultures for BAs control.

In summary, this study evaluated the BAs profiles of 16 typical Chinese sausage samples, indicating the potential BAs-related safety risk in Chinese sausages. Based on microbial community analysis results, 13 representative strains were selected from the dominant microbial genera, and their contributions to BAs formation and degradation were explained. Moreover, five *Staphylococcus* strains were confirmed to be efficient for BAs control in the fermented meat model. This study not only explained the microbial contribution to BAs accumulation in Chinese sausages, but also provided the potential starter cultures for BAs control in fermented meat products industry.

## Author Contributions

XW and LL designed the study. DZ and LR executed the experimental work. LL and DZ analyzed the data. XW contributed reagents and materials. LL, XW, ZW, SC, and LX wrote and revised the manuscript. All authors read and approved the final manuscript.

## Conflict of Interest Statement

LX was employed by the company Carollo Engineer, Inc. The remaining authors declare that the research was conducted in the absence of any commercial or financial relationships that could be construed as a potential conflict of interest.

## Footnotes

1 http://www.mothur.org/wiki/Schloss_SOP#Alpha_diversity

2 http://blast.ncbi.nlm.nih.gov/Blast.cgi
