# Supplementary material for: Evaluation of the Biogenic Amines and Microbial Contribution in Traditional Chinese Sausages
Source: Front Microbiol. 2019 May 3;10:872. doi: 10.3389/fmicb.2019.00872 (PMC6510162; doi:10.3389/fmicb.2019.00872)
Supplement: Supplementary file 1 [file Data_Sheet_1.docx]

# Supplementary Materials

**Table S1 Lists of primers to detect the genes involved in the formation of BAs.**

| Enzyme | Primer name | Sequence 5’-3’ | Expected amplicon size | Reference |
| --- | --- | --- | --- | --- |
| Histidine  decarboxylase | HdC1 | TTGACCGTATCTCAGTGAGTCCAT | 174 bp | ([Fernández et al., 2006](#_ENREF_14)) |
|  | HdC2 | ACGGTCATACGAAACAATACCATC |  |  |
| Tyrosine  decarboxylase | TD2 | ACATAGTCAACCATGTTGAA | 1100 bp | ([Coton et al., 2004](#_ENREF_6)) |
|  | TD5 | CAAATGGAAGAAGAAGTAGG |  |  |
| Ornithine  decarboxylase | ODF | CATCAAGGTGGACAATATTTCCG | 500 bp | ([Elsanhoty and Ramadan, 2016](#_ENREF_13)) |
|  | ODR | CCGTTCAACAACTTGTTTGGCA |  |  |
| Agmatine  deiminase | AgmSq1  AgmSq2 | CAAGATTTDTTCTGGGCHTTYTTCTC  TTGGHCCACARTCACGAACCCT | 700 bp | ([Ladero et al., 2011](#_ENREF_28)) |
| Agmatine  deiminase | AgD1  AgD2 | CAYGTNGAYGGHSAAGG  TGTTGNGTRATRCAGTGAAT | 600 bp | ([Coton et al., 2010](#_ENREF_7)) |
| Lysine  decarboxylase | Cad2F | CAYRTNCCNGGNCAYAA | 1185 bp | ([Landete et al., 2007a](#_ENREF_29)) |
|  | Cad2R | GGDATNCCNGGNGGRTA |  |  |
| Lysine  decarboxylase | CadAf | GCTGGGTTCACTGCTGGA | 220 bp | ([Guarcello et al., 2016](#_ENREF_18)) |
|  | CadAr | GGCGTAATGTAGCTCATCA |  |  |

**Table S2. BAs contents in model fermented meat product inoculated with different strains.**

| Chinese sausage sample | Tryptamine (mg/kg) | β-Phenethylamine (mg/kg) | Putrescine (mg/kg) | Cadaverine (mg/kg) | Histamine (mg/kg) | Tyramine (mg/kg) | Spermidine (mg/kg) | Spermine (mg/kg) | Total  (mg/kg) |
| --- | --- | --- | --- | --- | --- | --- | --- | --- | --- |
| Sp | 41.02±6.45 b | 370.88±70.47 bc | 108.11 ±6.48 a | 1194.54 ±159.55 c | 182.85±14.76 b | 410.24 ±111.61 ab | 13.63 ±0.75 cd | ND | 2321.28 ±370.07 b |
| Se | 12.90±3.49 a | 202.77± 26.77 a | 258.34 ±74.97 b | 292.51 ±62.84 a | 52.65 ±5.89 a | 317.46 ±36.56 a | 7.85 ±1.13 a | ND | 1144.49 ±211.65 a |
| Sc1 | 19.07 ±2.58 a | 213.41 ±15.37 a | 196.22 ±57.76 ab | 503.42 ±64.66 ab | 59.98±3.26 a | 411.22 ±62.46 ab | 10.21 ±1.08 abc | ND | 1413.54 ±98.86 a |
| Sc2 | 32.23 ±7.23 ab | 320.73± 71.34 ab | 268.08 ±65.78 b | 607.05± 65.20 b | 84.02± 2.56 a | 347.11± 91.65 a | 12.79 ±0.28 bcd | ND | 1672.01 ±85.27 a |
| Ss | 25.71 ±0.92 ab | 221.14 ±28.38 a | 184.08 ±13.61 ab | 734.91 ±16.05 b | 85.42 ±3.80 a | 413.42 ±38.02 ab | 10.00 ±1.03 ab | ND | 1674.68 ±101.81 a |
| CK | 62.49±14.05 c | 477.13± 56.06 c | 260.55 ±32.44 b | 1828.62± 70.98 d | 260.85 ±42.77 c | 574.75 ±64.34 b | 14.91 ± 2.43 d | 85.65 ±5.92 | 3564.96 ±288.99 c |

Different letters (a, b, c, etc.) indicate significantly different means at P < 0.05 (analysis of variance (ANOVA)).


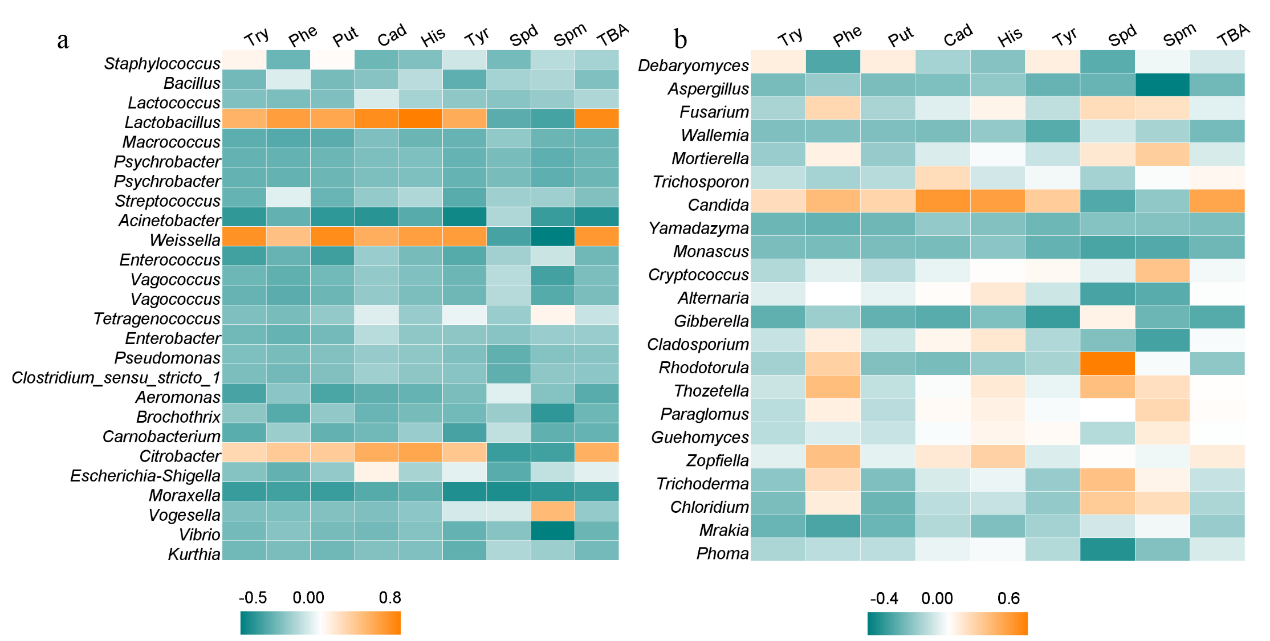


**Figure S1** Spearman analysis for correlation between biogenic amines and microbial communities at species level. (a) Bacteria, (b) Fungi.

**References**

Coton, M., Coton, E., Lucas, P., and Lonvaud, A. (2004). Identification of the gene encoding a putative tyrosine decarboxylase of *Carnobacterium divergens* 508. Development of molecular tools for the detection of tyramine-producing bacteria. *Food Microbiol.* 21**,** 125-130. doi: 10.1016/j.fm.2003.10.004

Coton, M., Romano, A., Spano, G., Ziegler, K., Vetrana, C., Desmarais, C., et al. (2010). Occurrence of biogenic amine-forming lactic acid bacteria in wine and cider. *Food Microbiol.* 27**,** 1078-1085. doi: [10.1016/j.fm.2010.07.012](http://dx.doi.org/10.1016/j.fm.2010.07.012)

Elsanhoty, R. M., and Ramadan, M. F. (2016). Genetic screening of biogenic amines production capacity from some lactic acid bacteria strains. *Food Control* 68**,** 220-228. doi: [10.1016/j.foodcont.2016.04.002](http://dx.doi.org/10.1016/j.foodcont.2016.04.002)

Fernández, M., Del Río, B., Linares, D. M., Martín, M. C., and Alvarez, M. A. (2006). Real-Time Polymerase Chain Reaction for Quantitative Detection of Histamine-Producing Bacteria: Use in Cheese Production. *J. Dairy Sci.* 89**,** 3763-3769. doi: [10.3168/jds.S0022-0302(06)72417-1](http://dx.doi.org/10.3168/jds.S0022-0302(06)72417-1)

Guarcello, R., De Angelis, M., Settanni, L., Formiglio, S., Gaglio, R., Minervini, F., et al. (2016). Selection of Amine-Oxidizing Dairy Lactic Acid Bacteria and Identification of the Enzyme and Gene Involved in the Decrease of Biogenic Amines. *Appl. Environ. Microbiol.* 82**,** 6870-6880. doi: 10.1128/aem.01051-16

Ladero, V., Rattray, F. P., Mayo, B., Martín, M. C., Fernández, M., and Alvarez, M. A. (2011). Sequencing and Transcriptional Analysis of the Biosynthesis Gene Cluster of Putrescine-Producing *Lactococcus lactis*. *Appl. Environ. Microbiol* 77**,** 6409. doi: 10.1128/aem.05507-11

Landete, J. M., De Las Rivas, B., Marcobal, A., and Muñoz, R. (2007a). Molecular methods for the detection of biogenic amine-producing bacteria on foods. *Int. J. Food Microbiol.* 117**,** 258-269. doi: 10.1016/j.ijfoodmicro.2007.05.001
